# Revealing sub-μm and μm-scale textures in H_2_O ice at megabar pressures by time-domain Brillouin scattering

**DOI:** 10.1038/srep09352

**Published:** 2015-03-20

**Authors:** Sergey M. Nikitin, Nikolay Chigarev, Vincent Tournat, Alain Bulou, Damien Gasteau, Bernard Castagnede, Andreas Zerr, Vitalyi E. Gusev

**Affiliations:** 1 LUNAM Universités, CNRS, Université du Maine, LAUM UMR-CNRS 6613, Av. O. Messiaen, 72085 Le Mans, France; 2 LUNAM Universités, CNRS, Université du Maine, IMMM UMR-CNRS 6283, Av. O. Messiaen, 72085 Le Mans, France; 3 LSPM, UPR-CNRS 3407, Université Paris Nord, Villetaneuse, France

## Abstract

The time-domain Brillouin scattering technique, also known as picosecond ultrasonic interferometry, allows monitoring of the propagation of coherent acoustic pulses, having lengths ranging from nanometres to fractions of a micrometre, in samples with dimension of less than a micrometre to tens of micrometres. In this study, we applied this technique to depth-profiling of a polycrystalline aggregate of ice compressed in a diamond anvil cell to megabar pressures. The method allowed examination of the characteristic dimensions of ice texturing in the direction normal to the diamond anvil surfaces with sub-micrometre spatial resolution via time-resolved measurements of the propagation velocity of the acoustic pulses travelling in the compressed sample. The achieved imaging of ice in depth and in one of the lateral directions indicates the feasibility of three-dimensional imaging and quantitative characterisation of the acoustical, optical and acousto-optical properties of transparent polycrystalline aggregates in a diamond anvil cell with tens of nanometres in-depth resolution and a lateral spatial resolution controlled by pump laser pulses focusing, which could approach hundreds of nanometres.

Knowledge of the pressure dependences of sound velocities and elastic moduli of liquids and solids at megabar pressures and the evolution of the texture of polycrystalline samples under compression is of extreme importance for a few branches of the natural sciences, such as condensed-matter physics, physics of the Earth and planetology, as well as for the monitoring and prediction of earthquakes and tsunamis and for nuclear weapons test control^1^,^2^. Experimental information on high-pressure material parameters, which can be measured at *P* > 25 GPa in laboratory conditions only using a diamond anvil cell (DAC), can be substantially influenced by spatial inhomogeneities and texture in polycrystalline aggregates^3^. It is well known that aggregate sound velocity depends on the characteristic dimensions of the individual crystallites, which are elastically anisotropic^4^, and that even a partial alignment of the crystallites i.e., orientational texture, biases the measured aggregate sound velocities^5^. Both of these factors prevent precise evaluation of the elastic moduli and require the development of experimental methods for the three-dimensional imaging of microscopic samples *in situ* when compressed in a DAC for the purpose of characterising both their morphological and orientational/directional texture.

In the past decade, significant progress has been achieved in imaging of isolated objects and multigrain bodies using two X-ray techniques. The first one, based on the spatial variation of X-ray absorption in chemically inhomogeneous samples, allows spatial resolution better than 100 nm^6^. The second one, based on X-ray diffraction (XRD), enables determination of grain position and orientation in a polycrystalline body with the spatial resolution approaching sub-μm level^7^,^8^. Both methods have provided the mentioned resolution only for samples recovered from high-pressure experiments^9^,^10^. However, compression of samples in a DAC limits the solid-angle region where the samples can be accessed by X-rays, and the weight of the DAC limits the accuracy of spatial adjustment, especially when sample rotation is needed. Degradation of spatial resolution for samples in a DAC is significant (on the order of 10 μm) when grains in polycrystalline aggregates are localised using the monochromatic XRD method^11^,^12^. By applying the polychromatic micro-diffraction method, a somewhat better resolution of 2–5 μm in the axial sample direction (or along the incident X-ray beam) was expected^13^. Lateral resolution on the order of 100 nm can be maintained for samples compressed in a DAC only if the resolution is defined by the size of the X-ray beam. For example, this is possible for the boundary between two homogeneous media with different absorption^14^ and for inhomogeneities (e.g., domains) if they are isolated or separated by distances significantly exceeding the beam size^15^. Very recently, Bragg coherent X-ray diffraction imaging was applied for the mapping of strains in an isolated single crystal of gold with a resolution of 30 nm, but only up to 6.4 GPa^16^. Despite all the progress, none of the above mentioned methods provides information on the elastic properties of the examined sample volume or of individual grains. In the present paper, we introduce an experimental method that overcomes this limitation and provides spatially resolved information (in both lateral and axial directions) on the elastic behaviour of optically transparent samples compressed in a DAC to pressures approaching 1 Mbar. This permits, in turn, *in situ* examination of the texture of a chemically homogeneous polycrystalline sample, provided the sample material is elastically anisotropic.

Of the methods for the evaluation of elastic parameters of condensed media at high pressures, an important role is played by those based on the interaction of laser radiation with acoustical phonons. Application of optical radiation is particularly suited for the DAC, in which the transparency of diamond, needed for optical access to the compressed sample, is essential. In the classic frequency-domain Brillouin scattering (BS) technique, frequency shifts of monochromatic light scattered by thermal (incoherent) acoustic phonons in the sample provide information on the velocities of the longitudinal and shear sound^17^,^18^,^19^. In laser ultrasonic techniques, light is employed not only for the detection of acoustic phonons, as in the classic BS, but also for their generation^20^,^21^. The probe light is predominantly scattered not by the thermal phonons but by coherent laser-generated phonons. The laser ultrasonic technique of impulsive stimulated Brillouin scattering (ISBS)^22^, based on the generation of monochromatic coherent acoustic waves by laser-induced gratings, has been applied at high pressures to measure the velocities of both bulk^23^,^24^ and interface^25^,^26^ acoustic waves. Recently, the picosecond laser ultrasonic technique^27^, based on both generation and detection of wide-frequency-band coherent acoustic pulses by femtosecond laser pulses, was first applied at high pressures in DACs^28^. The laser ultrasonic technique based on the generation of acoustic pulses by sub-nanosecond laser pulses and their detection by a continuous laser radiation is described in Ref. 29. In Refs. 28 and 30, transient optical reflectivity signals obtained by the technique of picosecond acoustic interferometry^31^, also called time-domain Brillouin scattering (TDBS) (see Methods and Fig. 1), were reported for the first time at high pressures. In an inhomogeneous medium, the TDBS signal at each time instance contains information on the local parameters of the medium in the spatial position of the laser-generated light-scattering acoustic pulse at this time instance^31^,^32^,^33^. Below, we report the application of TDBS to the examination of water ice in a DAC at pressures of 57 and 84 GPa, revealing the characteristic features of its micro-crystallinity.

Classic frequency-domain BS experiments with single-crystal H_2_O ice are extremely rare^34^,^35^ and have been reported only at relatively low pressures (below 10 GPa); experiments at higher pressures have been conducted on polycrystalline ice^34^,^36^,^37^,^38^. The problems in the application of the classic BS by thermal phonons in polycrystalline materials are well known^19^,^36^,^37^,^38^. The BS signal is very weak, and signal collection can take dozens of hours. The Brillouin spectral lines not only are considerably broadened^19^,^36^ but are also split^19^,^36^,^38^ because of the simultaneous contributions to the scattered light from multiple differently oriented crystallites, i.e., elastically anisotropic grains, inside the scattering volume. Detection of the broad Brillouin lines provides an opportunity to extract only the orientation-averaged, i.e., so-called aggregate, sound velocities^36^, whereas splitting of the Brillouin peaks requires data collection through a large number of different scattering volumes covering many randomly oriented grains, with the goal of establishing the maximum and the minimum boundaries of compressional and shear sound velocities^38^. The experiments with cubic polycrystalline ices are further complicated by their optical isotropy^39^, which makes it impossible to visualise/characterise the grain distribution, i.e., the polycrystalline aggregate texture, by birefringence, which could be very useful in the case of grains without inversion symmetry^34^,^40^. The in-depth spatial resolution in the classic BS microscopy^41^ applied to three-dimensional imaging^42^ currently exceeds tens of micrometres.

Here, we report experimental “visualisation” of the texture in a polycrystalline aggregate of H_2_O ice compressed in a DAC to pressures approaching 1 Mbar by TDBS. The demonstrated two-dimensional, in-depth and lateral, imaging of the texture in cubic, i.e., optically isotropic, ice is due mostly to the contrast provided by differences in sound wave velocities inside the differently oriented crystallites/domains relative to the propagation direction of the acoustic and probe-laser pulses. When a coherent acoustic pulse of nanometre to sub-micrometre spatial length and of lateral dimension controlled by pump laser focusing propagates inside the sample, we resolve in time/space with previously inaccessible precision sound velocities corresponding to the particular orientations of the spatial domains, in which the coherent acoustic pulse is currently localized.

## Results

**Figure 1:**
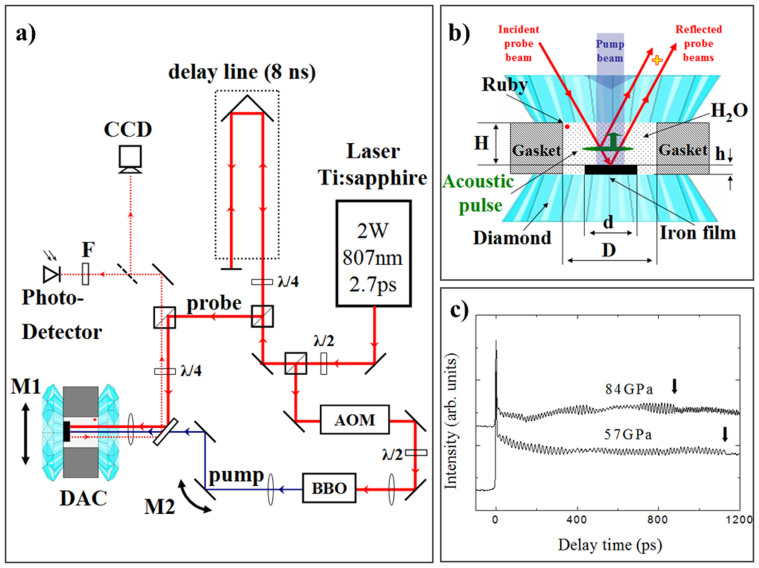
Time-domain Brillouin scattering in a diamond anvil cell. TDBS is an optical pump-probe technique in which the pump laser pulse generates in a light-absorbing opto-acoustic transducer a picosecond acoustic pulse that propagates through an optically transparent sample contacting the transducer while the probe laser pulse monitors the propagation of the acoustic pulse ((a) and (b)). The probe laser pulse, delayed in time relative to the pump laser pulse ((a) and (b)), preferentially interacts with those coherent GHz phonons of the acoustic pulse spectrum that satisfy the conservation of momentum in photon-phonon photo-elastic interactions^31^,^45^. Weak light pulses scattered by the acoustic pulse interfere at the photo-detector with the probe light pulses of significantly higher amplitude reflected from various surfaces/interfaces of the set-up. The modification of the optical reflectivity is proportional, in leading order, to the product of these two scattered light fields. Thus, a heterodyning of a weak field against a strong one is achieved. The transient reflectivity signal varies with time because the relative phase of the light scattered by the propagating acoustic pulse and reflected by immobile surfaces/interfaces continuously changes with time due to the variation in the spatial position of the propagating acoustic pulse. If the acoustic pulse propagates at a constant velocity, the amplitude of the signal changes in time in a sinusoidal manner at a GHz frequency precisely equal to the Brillouin frequency. Thus, measuring the period/frequency of this *time-domain* Brillouin oscillation provides information on the velocity of the acoustic wave in the sample. (a): Schematic presentation of the experimental setup. (b): Sample in a DAC with qualitative presentation of some of the probe optical rays contributing to the TDBS detection. For the characteristic dimensions of the ice sample and of the opto-acoustic transducer noted in (b), see Methods. (c): Transient reflectivity signals detected in the ice samples compressed in a DAC to 57 and 84 GPa as a function of the delay time between the probe and the pump laser pulses. Arrows indicate the times for the first arrival of the photo-generated acoustic pulse at the interface of ice and the diamond anvil.

The TDBS experiments on samples compressed in a DAC were conducted using the pump/probe configuration for transient reflectivity optical measurements presented in Fig. 1(a) (see Methods).

### Experimental signals and their processing

**Figure 2:**
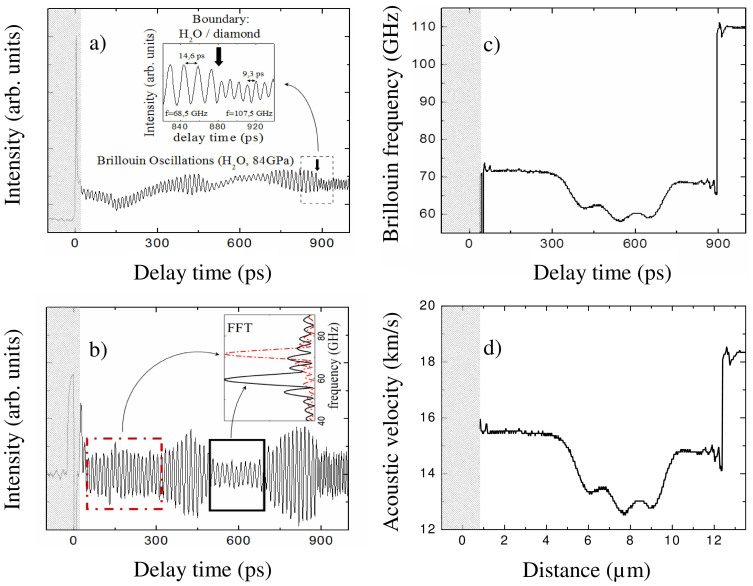
Revealing in-depth spatial inhomogeneity of H_2_O ice X at megabar pressures. (a): Typical time-resolved reflectivity signal in ice compressed in a DAC to 84 GPa. The vertical arrow marks the time of transmission of the laser-generated acoustic pulse across the interface of ice with diamond. Insert: zoom of the signal in the vicinity of the ice/diamond interface. (b) TDBS signal obtained by subtracting in signal (a) the time-varying thermo-reflectance contribution caused by transient heating of the sample. Insert: The Fourier spectra of the signal inside two temporal windows, marked by rectangles, demonstrate the shift of the Brillouin frequency from 60 GHz to approximately 73 GHz, indicating spatial inhomogeneity of the ice sample. For each time window, the obtained frequency value is an average over multiple crystallites in the volume from which the signal is collected. The lateral size of the probed volume is determined by the elliptical intensity correlation function with the axes of 3 μm and 4.5 μm FWHM for the pump and probe beams (see Methods). The cross-sectional area of the tested volume can be estimated as 10 μm^2^. The axial, i.e., in-depth, dimensions of the probed sample volumes are determined by the duration of the time windows and exceed, in this particular case, 3 μm. (c): Temporal profile of the Brillouin frequency of the TDBS signal shown in (b). The Fourier transform was performed in the rectangular moving temporal window of 100 ps duration, corresponding to the in-depth distance of approximately 1.5 μm. (d): Spatial variation of the longitudinal sound velocity obtained from the temporal dependence of the Brillouin frequency shown in (c) using the extrapolated refractive index values of ice^39^. The coordinate in (d) is evaluated by integrating the sound velocity over acoustic propagation time only in a part of the experimental window, which does not include short delay times (shadowed in Fig. 2), where the determination of the Brillouin frequency is not sufficiently exact because of imperfect filtering of the thermo-reflectance contribution at GHz frequencies. Thus, the distance in (d) should be measured relative to the ice/diamond interface.

Typical transient reflectivity signals recorded in the experiments at 57 and 84 GPa, where water ice appears most likely in phase X with ordered positions of protons^43^,^44^, are presented in Fig. 1(c). Here, the temporal window spans the delay times necessary for one-way propagation of the photo-generated coherent acoustic pulse from the iron opto-acoustic generator to the ice/diamond interface (see Fig. 1(b)). The arrival times of the acoustic echoes at the ice/diamond interface, indicated by arrows in Fig. 1(c), manifest themselves as an abrupt diminishing in the amplitude of the Brillouin oscillation at 880 ps and 1134 ps for 84 GPa and 57 GPa, respectively. Fig. 1(c) and, especially, the insert in Fig. 2(a) clearly show, even without any signal processing, that the transmission of the acoustic pulse across the ice/diamond interface is additionally accompanied by an abrupt increase in the frequency of the oscillating part of the transient reflectivity. These observations are in accordance with the estimated weak reflection, less than 15% in amplitude, of acoustic waves from the ice/diamond interface, the lower photo-elastic (acousto-optic) constants of diamond in comparison with those of ice at the optical probe wavelength, the larger Brillouin frequency shifts in diamond than in ice measured by the classic BS at similar pressures^36^ and the decrease in the acoustic pulse spectral amplitude with increasing frequency. This arrival time is known to provide information on the acoustic velocity averaged over the path of the coherent acoustic pulse and on the sample thickness^32^,^33^,^45^,^46^,^47^ (see Methods).

The non-monotonous variations in time of the Brillouin oscillation amplitude before the arrival of the coherent acoustic pulse at the ice/diamond interface, which are observable in Figs. 1(c), 2(a) and 2(b) without any signal processing, are a strong indication of the samples' spatial inhomogeneity, i.e., polycrystallinity and texturing. These variations cannot be attributed to the beatings of TDBS signals of different frequencies and are manifestations of texturing in the distribution of both photo-elastically (acousto-optically) and elastically anisotropic sub-μm grains composing the ice aggregate (see Section on the amplitude of the TDBS signal).

Further indication of the spatial inhomogeneities could be the variations with the delay time of the frequency of the Brillouin oscillation^31^,^32^,^33^. For the bulk of the ice X sample, this can be revealed by a Fourier transform performed inside an appropriately chosen time window after subtraction from the total time-resolved reflectivity signal of the time-varying thermo-reflectance contribution caused by sample heating^32^,^33^. This signal processing leads to the TDBS signal, which is due to the interaction of the probe light with the photo-generated acoustic pulse only (Fig. 2(b)). The plots in the insert of Fig. 2(b), as well as all other FFT plots calculated in this work, were clearly dominated by a single strong peak related to the Brillouin frequency of the longitudinal sound wave. The ±10% deviations of the Brillouin frequencies from their average, revealed here (insert in Fig. 2(b)), clearly indicate the presence of spatial elastic inhomogeneities in the ice sample due to polycrystallinity/texturing.

Performing a Fourier transform in a continuously moving smoothed time window of appropriately chosen duration and taking the frequency of the maximum in the spectra makes it possible to extract the temporal-profile of the dominant Brillouin frequency along the particular path of the coherent acoustic pulse propagation. Changing the time-window size allows different degrees of averaging along the sound propagation direction. The resolution of such in-depth profiling of the spatially inhomogeneous media using TDBS is known to be limited either by the spatial length of the coherent acoustic pulse or by restrictions introduced by specific signal processing techniques^32^,^33^. In our experiments, the duration of the emitted longitudinal acoustic pulse is controlled by a pump laser pulse duration of 1.9 ps, and its width in ice near the opto-acoustic generator is approximately 30 nm at 84 GPa (see Supplementary Note 1). The observed abrupt changes in the amplitude and frequency of the Brillouin oscillations upon the arrival of the acoustic pulse at the ice/diamond interface (Fig. 2 (a)) indicate that, although this pulse could be broadened by bulk high-frequency absorption and scattering, the duration of the acoustic pulse does not exceed the Brillouin period of approximately 15 ps, corresponding to a spatial scale of approximately 0.23 μm at 84 GPa. For the Fourier transforms in the present study, we used the windows whose FWHM exceed the Brillouin periods. Thus, we achieved depth-profiling with the same in-depth spatial resolution (controlled by the duration of the moving Fourier transform window) throughout the whole sample thickness via the simplest signal processing technique. In-depth spatial resolution controlled by the acoustic pulse duration, achievable by advanced methods of signal processing^32^,^33^, is beyond the scope of the present report. In Fig. 2(c), we present the temporal profile of the Brillouin frequency for the TDBS signal shown in Fig. 2(b) obtained with the moving rectangular time window of 100 ps, corresponding to an in-depth spatial resolution of approximately 1.5 μm.

In our polycrystalline ice sample, the photo-generated coherent acoustic pulse, which is initially launched normal to the Fe/ice interface and propagates parallel to the direction of the probe laser beam, could be refracted when crossing such plane interfaces between the grains, which are not parallel to the Fe/ice interface. Thus, in general, the coherent acoustic pulse propagates non-collinearly to the probe light. In this case, evaluation of the momentum-conservation triangle composed of the wave vectors of an acoustic phonon and of the incident and the scattered probe light photons leads to the following solution for the Brillouin frequency *f_B_* (see Refs. 32, 45):
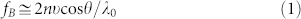


Here, *υ* denotes the speed of the acoustic wave along a particular direction of its propagation in elastically anisotropic media, *λ*_0_ is the wavelength of the probe light in vacuum, *n* is the optical refractive index of the media for the probe light, and *θ* is the angle between the propagation directions of the probe light and the sound. In general, three factors in equation (1), i.e., *θ*, *n* and *υ*_, _could be responsible for different values of the Brillouin frequency when the coherent acoustic pulse travels along its propagation path. We estimated theoretically and confirmed by additional experiments that variations of the refractive index play a negligible role in comparison with those of sound velocity (see Section on the induced optical anisotropy) In Section on the acoustic refraction and diffraction, we demonstrate theoretically that variations caused by the acoustic beam refractions could also be neglected in the first approximation. Thus, in the following analysis, we assume that the experimentally revealed variations in the Brillouin frequency are due to the variations of the sound velocity only, i.e., that they are dominantly caused by the different orientations of elastically anisotropic crystallites or groups of crystallites in the polycrystalline ice aggregate. As an example, assuming in equation (1) that *θ* = 0 and taking the refractive index of the ice from the literature^39^, it is a straightforward process to obtain from the dependence in Fig. 2(c) the dependence of the acoustic velocity in ice first on time and then on the in-depth coordinate (Fig. 2(d)).

**Figure 3:**
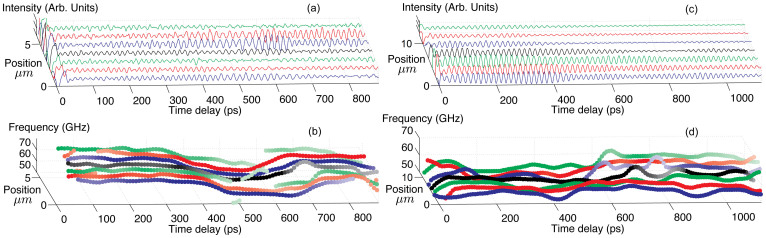
Revealing μm-scale texture in a polycrystalline H_2_O ice aggregate at megabar pressures via two-dimensional imaging by the time-domain Brillouin scattering technique with an in-depth spatial resolution of ~1 μm. (a): TDBS signals in H_2_O ice at 84 GPa, obtained by displacing the sample relative to the co-focused pump and probe laser beams in a lateral direction, i.e., parallel to the ice/diamond interface, by 1 μm steps. The spatial in-depth distance of 1 μm corresponds here to an ~66 ps delay time. (b): Two-dimensional images of the Brillouin frequency magnitude obtained by processing the signals in (a). (c) TDBS signals in H_2_O ice at 57 GPa, obtained by displacing the sample a in the lateral direction by 2 μm steps. The spatial in-depth distance of 1 μm corresponds here to an ~75 ps delay time. (d): Two-dimensional images of the Brillouin frequency magnitude obtained by processing the signals in (c). The isometric representation of the TDBS signals and of the temporal dependences allows better recognition of their changes with position on the sample. In (b) and (d), the signal amplitude is correlated with the symbol colour: the darker the symbol is, the higher is the maximum amplitude of the Brillouin signal, and vice versa. Image (b) reveals a clear, large-scale layering of the ice aggregate at 84 GPa in the direction normal to the diamond anvil culets. The thickness of the layers in this large-scale texture is approximately 3–5 μm. The image (d) of ice at 57 GPa reveals two regions separated lateraly, which exhibit a much less pronounced in-depth layering than at 84 GPa. Visually, the lateral separation in (d) occurs in the middle of the laterally scanned region. In addition, smaller scale inhomogeneities, with a thickness of approximately 1 μm, controlled by the intentionally reduced spatial resolution, are observed at some depth positions at both pressures.

The experimentally observed spatial inhomogeneity of the polycrystalline aggregates of ice X (Fig. 2(d)) can be more deeply characterised in two-dimensional imaging experiments, performing such TDBS measurements at several consecutive spatial positions along a lateral sample coordinate. In Fig. 3, to reveal large-scale inhomogeneities in the ice X aggregate, we show the Brillouin frequency distribution obtained with the in-depth spatial resolution of approximately 1 μm. The resolution is determined by a 67 ps FWHM duration of the Hanning temporal window chosen for the Fourier transform (see Methods). Fig. 3(b) and 3(d) show the time-resolved profiles of the Brillouin frequency, which we hereafter call “images”, obtained for the ice sample at 84 GPa and 57 GPa, respectively, by its displacement in a lateral direction.

The results presented in Fig. 3 clearly demonstrate the ability of the TDBS technique to reveal texturing of transparent polycrystalline aggregates such as our ice sample with a spatial scale better than 1 μm.

**Figure 4:**
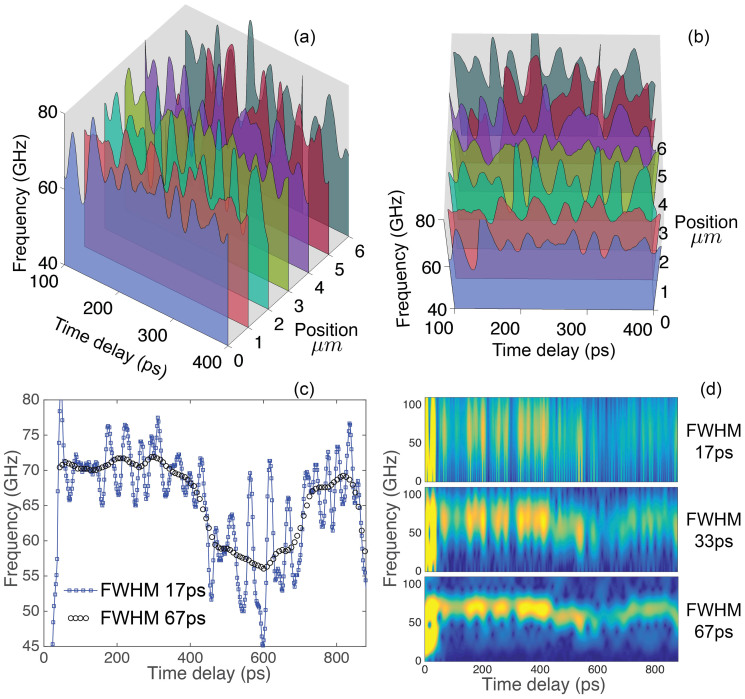
Revealing sub-μm-scale texture in a polycrystalline H_2_O ice aggregate at megabar pressures via two-dimensional imaging by the time-domain Brillouin scattering technique with an in-depth spatial resolution of ~0.26 μm. (a) and (b): Two-dimensional depth profiles of the Brillouin frequency in H_2_O ice at 84 GPa, obtained by displacing the sample relative to the pump and probe co-focused laser beams in a lateral direction, i.e., parallel to the ice/diamond interface. The profiles in the figure are measured at six particular positions shifted with respect to each other in the same direction by 1 μm. The size of the moving Hanning time window for the Fourier transform is 17 ps FWHM, providing the in-depth spatial resolution of 0.26 μm. While the 3D representation in (a) makes the spatial order of the measurements clear, the view in (b) of the same profiles provides a better understanding of both in-depth and lateral structuring. The colour code for the measured velocity profiles is the same in both images. The temporal images are presented starting at the 100 ps delay time to avoid the region of pulse propagation in the vicinity of the Fe photo-generator, where the results are commonly less precise because of non-perfect elimination of the high-amplitude thermo-reflectance contribution from the time-resolved reflectivity data. (c): Superposition of the short-scale texturing revealed in Fig. 4(a, b) with the coarse scale texturing revealed in Fig. 3(b). The variations of the Brillouin frequency are extracted from the TDBS signal accumulated at a lateral coordinate of 1 μm in Figs. 3(b) and 4(a, b). The maximum changes of the Brillouin frequency relative to the average level are approximately ±26%. (d): Time-frequency spectra of the selected signal obtained with different durations of the moving Hanning window.

The characteristic dimension of the individual crystallites in the ice X aggregate estimated from our XRD measurements at the considered pressures of 57–84 GPa is 0.45 μm (see Methods). To check the sensitivity of the TDBS technique to in-depth inhomogeneities at a sub-micrometre scale, we processed the signals in a moving Hanning temporal window of 17 ps FWHM duration, approximately corresponding to a single period of the Brillouin oscillation at 84 GPa. The expected spatial resolution of approximately 0.26 μm is still controlled by the duration of the moving window. The results of the processing of the TDBS signals (Fig. 3(a)) in the delay time interval from 100 ps to 400 ps are presented in Fig. 4, which shows strong variations of the BS frequency (up to ±26% for the maximal-to-minimal velocity variation, and up to ±10% for adjacent maxima and minima) and, accordingly, of the longitudinal sound velocity by moving in depth (corresponding to the delay-time axis). From theoretical calculations for ice X, the maximal-minimal velocity variation approaches, within a single crystal, ±16%, thus indicating that ice X should be strongly anisotropic^48^. However, the degree of anisotropy appears to be difficult to calculate theoretically, and no experimental information, except the present work, is available. The characteristic spatial (in-depth) scale of the BS frequency variations is 0.26–0.6 μm. We should bear in mind the relatively large cross section of ~10 μm^2^ of the opto-acoustically tested volume and the small size of the ice crystallites of ~0.45 μm, implying that the recorded BS signal (at any delay time) is averaged in both lateral directions over approximately 50 individual crystallites. A large change in the BS frequency by moving in depth to the next layer of grains (increase or decrease of the delay time by 20–30 ps) suggests a significant degree of crystallographic ordering in each of the layers but also a significant difference in the average crystallographic orientation of the adjacent ordered layers. This could be considered as a strong indication of sample texturing parallel to or inclined with respect to the anvil culets. However, we do not recognise any pattern by shifting our sample in a lateral direction (Fig. 4(a) and 4(b)), even though the step size of 1 μm implies a partial overlapping of the examined volumes of the adjacent depth profiles of the BS frequency. No pattern can also be recognised when the time-axes in Fig. 4 (a) and (b) are recalculated into in-depth coordinate axes. This observation excludes the above discussed possibility of sample texturing parallel to the diamond anvils, at least on the scale significantly larger than 1 μm, in the lateral directions. The only possible model that we can currently suggest is a sample that consists of or contains orientationally (or elastically) homogeneous mesoscopic crystallite groups in the form of lamellae, discs, or lenses having a lateral size of approximately 1 μm and a thickness of 0.3–0.6 μm. Formation of such elastically homogeneous crystallite groups having only one distinct direction appears possible because in two crystallographic directions, [110] and [111], the sound velocities in ice X are predicted to be similar (only approximately ±3% different) and significantly higher than in the [100] direction (±16%). The plastic behaviour of ice X at the pressures of our experiment is also predicted to be anisotropic and dominated by only one slip system, namely, <111>{110}^48^. Finally, the particular lens-shaped or lamellar objects inside a polycrystalline sample compressed in a DAC have been reported in the literature for other solids, e.g., by Burnley et al.^49^.

In the suggested texture model, the signal generated in the spot of ~10 μm^2^ stems from only 10–13 mesoscopic groups, where each of the groups is elastically homogeneous (at least in the direction of the sound propagation), but the groups could be oriented differently with respect to one another. In another model, a smaller number of such groups could be embedded in a disordered polycrystalline matrix. In both cases, a high degree of averaging of the total Brillouin frequency is less probable owing to the small number of differently oriented mesoscopic groups. Consequently, large shifts in the frequency by moving through the sample are feasible. We emphasise that the adjacent maxima and minima in Figure 4 (for the 17 ps window) typically show variations on the order of ±10%, which are significantly below the maximal variation of ±26%. The estimated number of the mesoscopic objects of approximately 10 would be consistent with such variations. Thus, the measurement with a small time window of 17 ps could have revealed some degree of texturing of our ice sample compressed to 84 GPa at sub-μm scale. However, the ordered/coherent regions are small and do not extend in lateral directions significantly more than 1 μm. Note that the characteristic direction of texturing, parallel to the diamond culets, recognised on the large scale in Figs. 3(b) and 4(c) and on the short scale in Fig. 4, appears to be plausible if we consider the geometry of the sample and the uniaxial compression of the sample confined in the gasket.

Confirmation by TDBS scattering of the sub-μm size of individual crystallites provides us the opportunity to make a general statement that the averaging in our present experiments is due not only to the large number of crystallites scattering probe light in the tested volume but also to the strong diffraction of the light inside the scattered probe beam on its way to the detector. Actually, for the wavelength of the probe light of approximately 0.44 μm in ice X at 84 GPa, a part of the probe laser beam scattered in a grain with a diameter not exceeding 0.45 μm will diffract at distances shorter than 0.6 μm. We estimate here the diffraction length by the ratio of the surface area of the scatterer to the probe laser wavelength in the compressed sample. Consequently, the corrugations of the probe wave front caused by its scattering at the corrugated front of the coherent acoustic pulse propagating simultaneously in multiple crystallites is effectively smoothed by the diffraction at a sub-μm scale. Thus, the detected signal provides information on the phase variation of the smoothed front of the scattered probe light and, consequently, on the diffraction-averaged Brillouin frequency.

The above presented results show that a three dimensional imaging of the texture of a sample compressed in a DAC is feasible. Below we demonstrate that the influence on the obtained images of the optical anisotropy and of the off-axis propagation of acoustic waves can be disregarded.

### On the induced optical anisotropy

**Figure 5:**
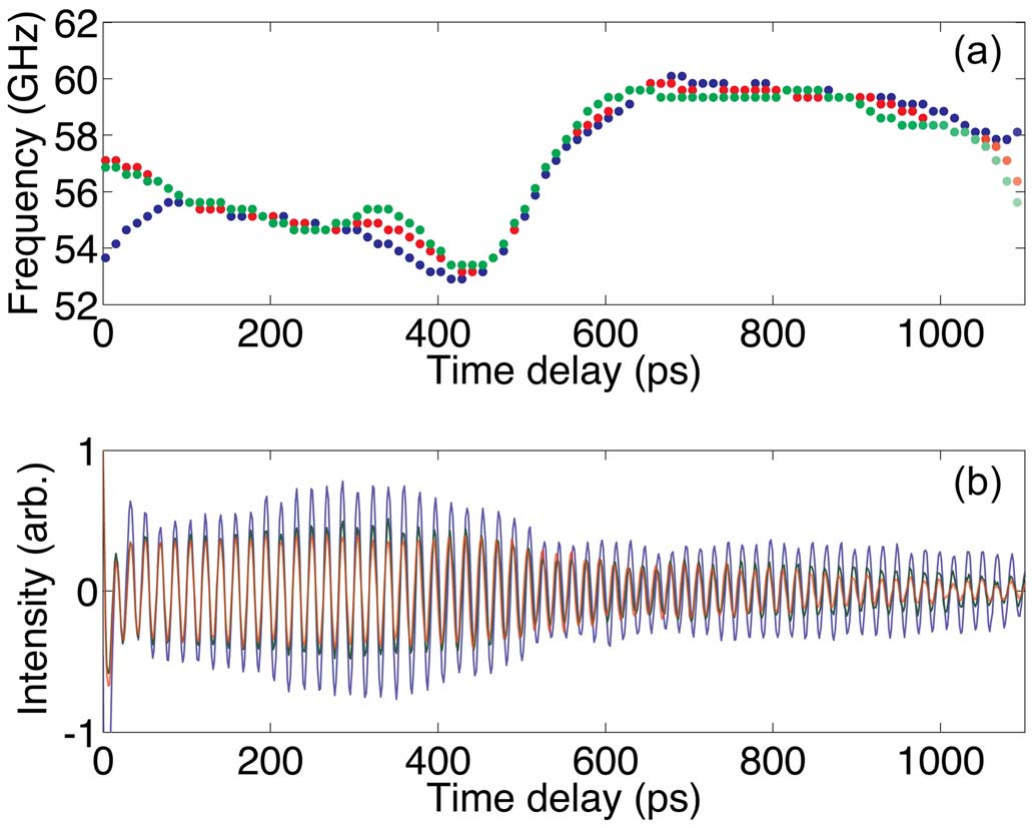
Revealing optical anisotropy of the cubic polycrystalline ice aggregate in a diamond anvil cell induced by non-hydrostatic loading. (a): Variations of the Brillouin frequency with time delay extracted from TDBS signals presented in (b), which were detected at 57 GPa for the probe light pulses of three different linear polarisations rotated in steps of 60°. Differences in the curves in (a) indicate that polarisation-dependent effects are present in our experimental system. However, there could be several physical phenomena contributing to the variation of the Brillouin frequency with the polarisation of the probe light in (a) (see Supplementary Note 2). In particular, stress-induced optical anisotropy of both the ice aggregate and the diamond anvil could be potentially involved. Although we are currently not able to identify unambiguously the physical reasons for the polarisation-dependent effect visible in (a), we believe this experimental test clearly indicates the weakness of the effect because the relative changes of the Brillouin frequency do not exceed 2%. Thus, possible variations of the refractive index of ice due to induced optical anisotropy cannot be responsible for the ±10% variations in the Brillouin frequency revealed in Figs. 2 and 4 and could be neglected when using equation (1) for the estimates of the acoustic velocity.

In optically anisotropic grains, the light experiences double or triple refraction and the optical rays, characterised by different refractive indexes, propagate after refraction with different velocities, which, in addition, depend on the direction of rays propagation relative to crystallographic axes^50^,^51^. However, the ice phases VII and X examined here are cubic and thus optically isotropic. Consequently, variations of the refractive index *n* could be caused in our samples only by anisotropy induced by nonhydrostatic stress component, i.e., due to uniaxial loading^52^. Our order of magnitude estimates (see Supplementary Note 2) demonstrate that induced optical anisotropy could cause variations of the refractive index of Δ*n*/<*n*>∝ 5·10^−4^, which are negligible in our experiments. Here, chevrons 〈…〉 denote average values. However, in view of the complexity of the phenomena of stress-induced anisotropy and the lack of data on the photo-elastic tensors of the H_2_O ices VII and X, we conducted additional test experiments with variable linear polarisation of the probe beam. The results presented in Fig. 5 confirm that optical anisotropy is induced in our system, but it is weak, and thus the variations of the refractive index in equation (1) could be disregarded.

### On acoustic refraction and diffraction

The maximal expected deviations in the direction of the coherent acoustic pulse propagation are estimated using acoustic ray theory in Supplementary Note 3. These estimates predict that the dominant cause of the relative variations of the Brillouin frequency, |Δ*f_B_*|/〈*f_B_*〉, in our experiments is the sound velocity variations, |Δ*υ*|/〈*υ*〉∝|Δ*f_B_*|/〈*f_B_*〉 ≤ 0.2, and the dependence on cos*θ* in equation (1) can be disregarded in the first approximation because Δ(cos*θ*)/〈cos*θ*〉 ≤ 0.04.

In our opinion, there is an additional and more important factor, specific to micro-crystalline samples, that could significantly diminish the maximum effective angles that should be used in the estimates based on equation (1). In fact, when the acoustic pulse crosses a layer of differently oriented crystallites, distributed laterally, the phase front of the acoustic pulse has a tendency to corrugate because of the difference in the magnitudes and the directions of the sound velocity in different crystallites. However, this tendency is strongly suppressed by the diffraction phenomena smoothing the amplitude and phase differences between the different parts of the acoustic beam. The shortest (30 nm) acoustic pulse, realised in our experiments in the vicinity of the Fe opto-acoustic generator (see Supplementary Note 1), corresponds to the characteristic acoustic wavelength of 0.2 μm (in ice X at 84 GPa). Consequently, the lateral corrugations of the acoustic pulse, which could accumulate in its refraction inside the crystallites with dimensions below 0.45 μm, are continuously washed out by diffraction at distances below 1.2 μm. Thus, while the diffraction of the total acoustic beam plays a negligible role in our experiments, the diffraction inside the acoustic beam supports the propagation of all parts of the beam quasi-collinear to the DAC axis.

### On the amplitude of the TDBS signal

**Figure 6:**
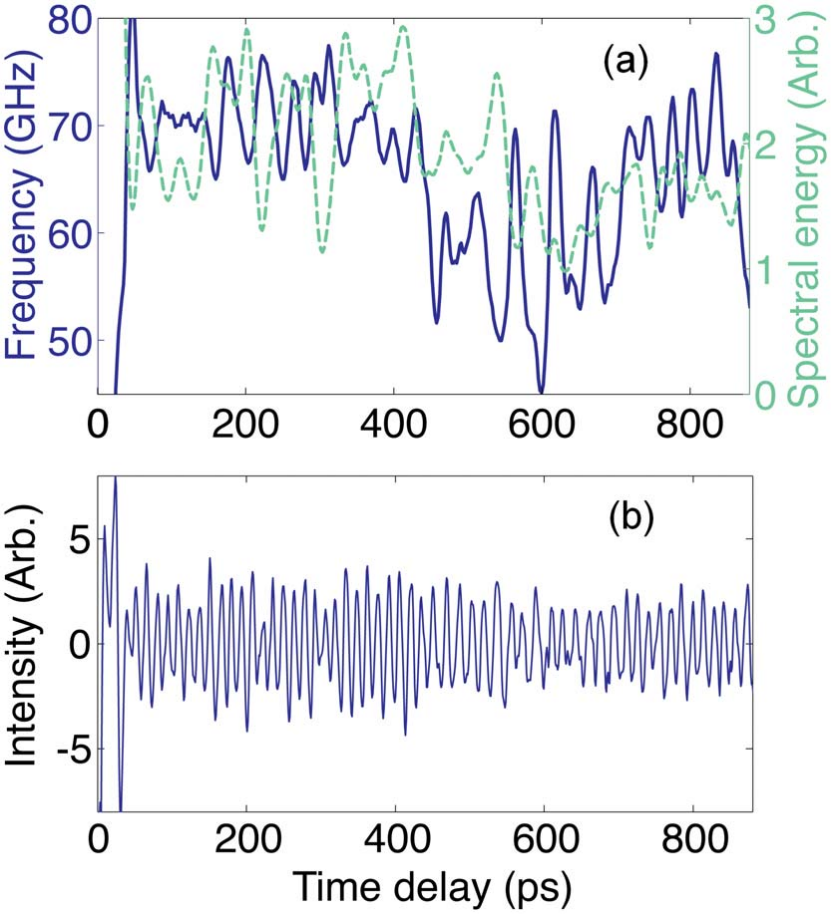
Correlation in temporal dynamics of the amplitude and frequency of the TDBS signal in ice X at 84 GPa accompanying the in-depth variations in the orientations of crystallites or their groups. (a): Simultaneous presentation, for comparison, of the temporal dynamics of the amplitude (green dashed line) and the frequency (blue solid line), determined for a Brillouin oscillation signal measured at a particular point of the sample at 84 GPa and shown in (b). The resolution is determined by a 17 ps FWHM duration of the Hanning temporal window chosen for the Fourier transform. The results presented in (a) definitely demonstrate the expected correlation between the variations of the frequency and the amplitude at a short sub-μm spatial scale, confirming that the physical origin of both is in the orientational texture of the ice sample. Note that variations of frequency and amplitude are mostly in anti-phase. The correlations between the frequency and the amplitude at a larger, i.e., μm, spatial scale are obscured by the influence on the TDBS signal amplitude of the absorption/scattering of both the coherent acoustic pulse and the probe light.

The discussion in Supplementary Note 4 indicates that non-monotonous variations of the TDBS signal amplitude in time in our experiments (see Figs. 1 (c) and 3 (a) and (c), for example) are not the manifestation of beatings/interference among different frequency components of the signal but instead are the second (in addition to the Brillouin frequency variation in time) direct manifestation of the misorientation of grains or groups of grains in the examined polycrystalline H_2_O ice aggregates. Actually, cubic crystallites of ice are optically isotropic but anisotropic photo-elastically (acousto-optically)^51^,^53^. The magnitude of the effective photo-elastic constant, which couples collinearly propagating sound and light, depends on the direction of their propagation inside individual crystallites. The amplitude of the TDBS oscillation is expected to be different in the differently oriented crystallites relative to the sample normal. Fig. 6 compares the measured variations of the amplitude and frequency, revealing their coherent dynamics at the sub-μm spatial scale; the variations of the amplitude and the frequency are mostly occurring in anti-phase. These experimental observations strongly indicate that the physical origin of both is in the orientational texture of the crystallites in our sample.

Because the amplitude *A* of the Brillouin oscillation is directly proportional to the effective photo-elastic constant and inversely proportional to the square root of the acoustic velocity, (*A* ∝ *pn*^−1^(*υρ*)^−1/2^, where *p* and *ρ* denote the photo-elastic constant and density, respectively)^32^,^54^, it could be expected that the photo-elastic anisotropy provides a dominant contribution in comparison with the elastic anisotropy to the non-monotonous variations of the amplitude of the Brillouin oscillations in polycrystalline aggregates. Our estimates, using the data in Fig. 6, demonstrate that the magnitude of the amplitude variations significantly exceeds that expected due to the variations of the acoustic velocity only, i.e., Δ*A*/<*A*>∝ −(1/2)(Δ*υ*/<*υ*>) ∝ −(1/2)(Δ*f*/<*f*>). This indicates an important role of acousto-optic (photo-elastic) anisotropy.

## Discussion

In this work, we were able to approach a high in-depth resolution of approximately 0.26 μm in H_2_O ice samples compressed in a DAC to 56 and 84 GPa. This value is significantly below the estimated average crystallite size in these samples. This result can be achieved for any other transparent material compressed in a DAC to similar or higher pressures. Currently, no other technique can provide such a detailed visualisation of a polycrystalline aggregate microstructure of a chemically homogeneous material compressed in a DAC, especially along the axial direction. Even the simplest signal processing, applied in the present work for processing of the TDBS signals, provides access to characterisation both of the in-depth dimensions of the micro-crystallites and of the texturing of the polycrystalline ice aggregates at different spatial scales from 0.26 μm, controlled by signal processing, to approximately 10 μm, controlled by the total thickness of the ice layer.

From the experimental geometry used in the present work, it follows that even when the shortest moving time window for the Fourier transform of 17 ps is used, the collected TDBS signal and the derived frequency/sound velocity are an average over approximately 50 grains. This is because the grain size was estimated to be approximately 0.45 μm, the lateral surface area tested by overlapping the probe laser beam and the coherent acoustic beam is approximately 10 μm^2^ and the wavelength of the probe laser light when propagating in ice at Mbar pressures is approximately 0.44 μm. Thus, it is difficult to access in the present sample and experiment geometry, at any position in the sample, the extreme sound velocity values, corresponding to the specific directions in a single crystal of ice X. The highest sound velocity observed in the present work by scanning through the sample will be lower than the maximal possible sound velocity in a single-crystal ice X (along the [111] direction of a cubic crystal). Similarly, the lowest sound velocity detected here represents the upper bound for the lowest possible sound velocity in a single-crystal ice X (along the [100] direction of a cubic crystal). One way to approach the limiting sound velocity values would be to use coarse powder or single crystals as starting samples. The grains could fracture under pressure due to nonhydrostatic loading or after phase transitions, but the fragments could remain sufficiently large to deliver to the detector the TDBS signals corresponding to individual grains. In the particular case of water, single crystals of ice VI and VII can be easily obtained via a slow compression at approximately 1 GPa when crossing the melting curve, e.g., as described in Refs. 35 and 55. Another way involves a significant improvement in the lateral resolution of the experimental set-up below 0.45 μm by application of advanced focusing methods^56^. Combined with the already available high in-depth resolution, the improved lateral resolution would enable determination of the limit values of sound velocities as well as a 3D visualisation of a transparent polycrystalline aggregate microstructure (from grain to grain) at megabar pressures.

The time-domain BS technique could have other advantages, in addition to improved spatial resolution, in comparison with the classic frequency-domain BS technique. The applicability of classic BS is substantially reduced and could even be impossible when the Brillouin spectral lines of the sample overlap with the Brillouin lines from the diamond anvils or from the pressure transmitting medium^24^,^57^, which are inevitably simultaneously detected for samples compressed in a DAC. This problem does not exist for the time-domain BS technique because the scattering of light by coherent acoustic phonons takes place in the sample only, well before the photo-generated acoustic pulse reaches the sample/diamond or sample/pressure-medium interface. Moreover, the TDBS allows direct comparison of the sound velocities and/or elastic moduli of two or more sample materials placed simultaneously in the high-pressure volume of a DAC. This comparison could be of use by establishing an absolute pressure scale at Mbar pressures.

## Conclusions

The TDBS-based imaging technique described herein provides for each crystallite (or group of crystallites) in chemically homogeneous transparent aggregate usable information on its orientation (if the material is elastically anisotropic) as well as on the value of the elastic modulus along the direction of the sound propagation. This extends the basis for successful application of highly developed micromechanical models of solid deformation at Mbar pressure. Over the long term, such experiments extended to Earth's minerals and high or low temperatures could ensure significant progress in understanding the convection of the Earth's mantle and thus the evolution of this and other planets.

The two-dimensional imaging of the polycrystalline aggregate in-depth and in one of the lateral directions reported here indicates the feasibility of three-dimensional imaging of transparent samples compressed in a DAC with a previously inaccessible resolution of tens of nanometres in-depth. The lateral spatial resolution is controlled by the pump and the probe laser pulse focusing. In perspective, improved signal processing of such TDBS data should provide an opportunity to follow the evolution of several Brillouin frequencies in the time domain, revealing simultaneous propagation of the coherent acoustic pulse across several mutually misoriented crystallites. In the future, TDBS experiments, conducted with both longitudinal and shear^58^,^59^,^60^ coherent acoustic pulses at several angles of probe light incidence^32^,^46^,^47^, could enable the determination of the spatial positions, dimensions and orientation, i.e., morphological and orientational texture, of the optical refractive index and all elastic moduli of the individual grains inside polycrystalline transparent aggregates compressed to megabar pressures.

## Methods

### Time-domain Brillouin scattering

In the time-domain Brillouin scattering technique, which is also called picosecond acoustic interferometry and is a particular optical pump-probe technique, the pump laser pulse generates in a light absorbing opto-acoustic transducer a picosecond acoustic pulse that propagates through a sample contacting the transducer. Because a typical length of the picosecond acoustic pulse is on the nanometre to sub-micrometre spatial scale, the technique is perfectly suitable for the examination of materials confined in DACs, where sample sizes are typically several tens of micrometres to a few micrometres and grains in polycrystalline samples are typically less than 1 μm (Fig. 1 (b)). In the case of an optically transparent material, the probe laser pulse, delayed in time relative to the pump laser pulse (Fig. 1 (a) and (b)), preferentially interacts with those coherent GHz phonons of the acoustic pulse spectrum that satisfy the momentum conservation law in photon-phonon photo-elastic interactions, i.e., satisfy the BS condition. Weak light pulses scattered by the acoustic pulse interfere at the photo-detector with the probe light pulses of significantly higher amplitude reflected from various surfaces and interfaces of the set-up, such as the interfaces of the sample with the diamond and of the opto-acoustic transducer with the sample (Fig. 1 (b)). The detected modification of the transient optical reflectivity is proportional, in leading order, to the product of these two scattered light fields. Thus, a heterodyning of a weak field against a strong one is achieved in picosecond ultrasonic interferometry. The measured transient reflectivity signal varies with time because the relative phase of the light scattered by the propagating acoustic pulse and reflected by immobile surfaces/interfaces continuously changes with time due to the variation in the spatial position of the propagating acoustic pulse. If the acoustic pulse propagates at a constant velocity, i.e., in a spatially homogeneous medium, the phase difference between the interfering light fields linearly changes in time and, as a consequence, the amplitude of the signal changes in time in a sinusoidal manner at a GHz frequency precisely equal to the Brillouin frequency^31^. Thus, measuring the period/frequency of this time-domain Brillouin oscillation provides information on the velocity of the acoustic wave in the sample. In the collinear scattering geometry of the TDBS experiments^28^,^30^, the Brillouin frequency is proportional to the product of the sound velocity and the optical refractive index of the sample at the probe wavelength (see Eq. (1) in Results).

In an inhomogeneous medium, the TDBS signal at each time instance contains information on the local parameters of the medium in the spatial position of the laser-generated light-scattering acoustic pulse at this time instance^31^. It has previously been demonstrated, although under ambient conditions, that this effect can be used for the depth-profiling of inhomogeneous transparent media with nanometre-scale resolution limited by the spatial length of the laser-generated coherent acoustic pulse^32^,^33^.

### Diamond anvil cell and the samples

High pressures up to 84 GPa were generated via compression of samples between bevelled diamond anvils of Boehler-Almax design having a culet size of 300 μm mounted in a Boehler-Almax Plate DAC^61^. A hole in the centre of a pre-indented stainless steel gasket represented the sample volume filled with ice, and a thin iron foil in contact with one of the anvils and the sample. The iron foil served as the opto-acoustic generator for launching coherent acoustic pulses into the ice. A magnified schematic of the sample arrangement in the DAC is presented in Fig. 1 (b). The sample dimensions in the experiments conducted at 57 GPa and 84 GPa were, respectively, 103 μm and 90 μm in diameter *D* and 14.4 μm and 13.5 μm in thickness *H*. The diameters *d* of the iron opto-acoustic generators were 66 and 40 μm, respectively, and their thicknesses were approximately 2 μm at ambient pressure, in both experiments. Pressure was determined from the wavelength of the R_1_ fluorescence line of ruby grains distributed throughout the ice sample, whose red shift with pressure was calibrated earlier^62^.

### Pump and probe optical setup

The experiments on ice compressed in the DAC were performed using a typical pump/probe configuration for transient reflectivity optical measurements (Fig. 1 (a)) involving a pulsed Ti:Sapphire laser with the following characteristics: 2 W average power, 808 nm wavelength and 2.7 ps FWHM duration of the laser pulses at the repetition rate of 80 MHz. This radiation was divided by a polarising cube in the pump and probe beams. The pump laser beam was modulated acousto-optically at a frequency of 161.1 kHz for the subsequent realisation of the synchronous detection of the probe laser radiation scattered by the sample. Then, it was frequency-doubled by a 1 cm-long BBO non-linear crystal to obtain 25 mW of 404 nm wavelength light pulses of 1.9 ps duration at FWHM for the generation of coherent acoustic pulses in the Fe-foil near its interface with H_2_O ice. A computer-controlled optical delay line with a two-passage configuration allowed the introduction of the delays in the probe laser pulses relative to the pump laser pulses in an interval of 0–8 ns. The time-delayed probe laser pulses, for time-resolved detection of the BS induced by the coherent acoustic pulse, were at the fundamental wavelength of 808 nm of the laser. The photo-acoustic signals were obtained with time steps of 1.5 ps. Both pump and probe radiation were focused by a 50×-objective lens (numerical aperture 0.5, working distance 10.5 mm) on the DAC from the same side, and the scattered probe radiation was also gathered from the same side. The system of optical imaging, including a web-camera and the source of white light, was installed for the visualisation of the laser spots and the surface of the sample in the DAC. The pump laser beam was focused on the Fe/ice interface into an elliptical spot, whose dimensions, 9 μm in the horizontal direction and 5 μm in the vertical direction, were determined at the FWHM of the intensity of the image obtained by the camera. The probe laser pulse was focused into a circular spot. The pump spot was scanned relative to the probe in the vertical lateral direction along the Fe/ice interface perpendicular to the long axis of the ellipse. The scan was achieved using a variation in the angle Δθ of the incidence of the pump beam, provided by the rotation of a dielectric mirror installed on the computer controlled support (M2 in Fig. 1 (a)). The displacement Δ*x* of the pump beam on the surface of Fe is the following: Δ*x* = [*f*−*h*(1−1/*n*)]Δ*θ*, where *f* = 4 mm is the focusing distance of the objective, and *h* = 2.5 mm and *n* = 2.4 are the thickness and the refractive index of diamond, respectively. The position of the delay line was fixed at the maximum of the amplitude of the transient thermo-reflectance signal, which corresponds to the coincidence in time of pulses of the pump and probe beams. Then, the pump beam was scanned in the vertical direction to obtain the amplitude of the transient thermo-reflectance signal as a function of the position. This function, showing the correlation of the pump and probe beams in the vertical direction, provided the width of 3 μm at FWHM. This indicates a 4 μm radius of the probe laser focus and a 4.5 μm FWHM of the correlation function of the two beams in the direction of the long (horizontal) axis of the pump laser elliptical focus. In addition, the DAC was mounted on a motorised linear stage (M1 in Fig. 1(a)), allowing displacement of laser spots in the horizontal lateral direction on the surface of the opto-acoustic generator with a precision of 0.1 μm. The filter (F in Fig. 1 (a)) was introduced before the photo-detector to avoid its illumination by the pump radiation scattered from the sample.

### Processing of TDBS signals

Panels (b) and (d) in Fig. 3 represent the dominant frequency of the reflectivity signals as a function of time. The dominant frequency values at each time are obtained from a spectrogram analysis of the temporal signals with a Hanning weighted window of 67 ps (64 points at a 0.9595 THz sampling frequency). For each central time of this sliding analysis window, every ~8 ps, the spectral component with the maximum amplitude is extracted. The frequency determination precision is improved by interpolating the spectrum with a spline function, which provides frequency steps of 0.2 GHz. The maximum amplitude is used for the colour scale of the plotted symbols. Thus, the darker the symbol is, the larger is the maximum amplitude of the Brillouin signal, and vice versa.

### Estimates of the crystallite dimensions from X-ray diffraction data

We collected two-dimensional XRD patterns of the H_2_O ice VII and ice X samples compressed in a DAC using monochromatic synchrotron radiation of the beam-line P2.02 (Petra III, HASYLAB, DESY)^63^. The patterns were collected from sample areas of approximately 15 × 15 μm^2^; the sample thickness was also approximately 15 μm. The samples were rotated by approximately 4 degrees around the vertical axis to increase the number of grains in the Bragg diffraction condition. These measurement conditions led to a smooth distribution of intensity, without any gaps, along the diffraction rings for all observed *hkl* reflexes, which suggests a relatively large number of randomly oriented crystallites covering the full solid angle 4π. To estimate the smallest number of grains leading to smooth diffraction rings, we needed to know the solid angle covered by a single grain under the present experimental conditions. To determine this angle, we used the divergence angle of the X-ray beam, focused on our sample in a DAC by compound reflective lenses (CRL)^64^. The angle was estimated to be approximately 0.32 mrad using the information available on the web-site of the synchrotron beam-line^65^: The CRL had an acceptance of 400 μm and was located 1.2 m from the sample. The effective divergence, however, is larger, approaching approximately 0.8 mrad, because the diffracted spot on the 2D detector, located at the distance of 500 mm from the sample, is similar to the detector pixel size of 0.2 mm. By combining the effective divergence with the angle of rotation around the vertical axis, we determined the solid angle covered by diffraction from a single grain to be 5.6·10^−5^. Taking into account the cubic symmetry of the crystal structure of ice VII and ice X, we estimated the number of crystallites needed to cover the 4π solid angle by uniform XRD rings to be approximately 37,000. Accounting for the sample volume illuminated with the X-ray beam (see above), we estimated the average size of crystallites in our samples at 57–84 GPa to be ≤0.45 μm. This value is close to the dimensions of regions with a similar magnitude of the Brillouin frequency that we revealed by processing the TDBS data in the moving time window of 17 ps (Fig. 4).

### Measurements of aggregate and envelope sound velocities

We applied optical interferometry to determine the product *H*·*n* of the ice layer thickness between Fe/ice and ice/diamond interfaces *H* (see Fig. 1 (b)) and the optical refractive index *n* of H_2_O ice at the probe beam wavelength. Then, using the values of *n* extrapolated from the published data^39^, we estimated *H*. We measured the propagation times of the acoustic pulse between the Fe/ice and ice/diamond interfaces at each pressure in three different positions of the sample via determination of the time moment when the abrupt change in the Brillouin oscillation amplitude occurred, estimated three averaged velocities and found their average value. Thus, the estimated sound velocities at 84 GPa and 56 GPa are 15500 m/s and 13500 m/s, respectively, which are in a very good agreement with the values of the aggregate sound velocities of ice measured by classic BS^35^. We also applied the so-called envelop method^38^ to determine the C_11_ modulus in our samples. For this, we found the minimum value of the Brillouin frequency from the data detected at three different points of the samples and determined the minimal longitudinal wave velocity using *n* extrapolated from Ref. 39. The minimum velocity in cubic crystallites is along the [100] direction and depends only on C_11_ and the density of ice. Taking the values of densities reported in Ref. 44, we found C_11_ = 480 GPa at 84 GPa and C_11_ = 350 GPa at 56 GPa, in reasonable agreement with the values of 540 GPa and 350 GPa, respectively, extracted by classical BS^36^. A detailed report on the sound velocities of ice VII and X as a function of pressure will be presented in a separate paper.

## Author Contributions

V.E.G., N.C., A.Z., S.M.N., A.B. and B.C. designed the research. S.M.N. and A.Z. prepared the samples. S.M.N., N.C., A.Z. and A.B. contributed to the experiments. V.T., S.M.N. and D.G. performed signal processing. V.E.G., A.Z., D.G., S.M.N. and N.C. contributed to developing the theory. V.E.G., A.Z., S.M.N., N.C. and V.T. analysed and interpreted the experimental observations. V.E.G., A.Z., S.M.N. and N.C. wrote the manuscript.

## Supplementary Material

Supplementary Information Revealing sub-μm and μm-scale textures in H_2_O ice at megabar pressures by time-domain Brillouin scattering
