# Supplementary material for: Revealing sub-μm and μm-scale textures in H2O ice at megabar pressures by time-domain Brillouin scattering
Source: Sci Rep. 2015 Mar 20;5:9352. doi: 10.1038/srep09352 (PMC4366861; doi:10.1038/srep09352)
Supplement: Supplementary Information — Revealing sub-μm and μm-scale textures in H2O ice at megabar pressures by time-domain Brillouin scattering [file srep09352-s1.doc]

**Revealing sub-μm and μm-scale textures in H_2_O ice at megabar pressures by time-domain Brillouin scattering**

Sergey M. Nikitin^1^, Nikolay Chigarev^1^, Vincent Tournat^1^, Alain Bulou^2^,

Damien Gasteau^1^, Bernard Castagnede^1^, Andreas Zerr^3^,

and Vitalyi E. Gusev^1^

^1^ LUNAM Universités, CNRS, Université du Maine, LAUM UMR-CNRS 6613,

Av. O. Messiaen, 72085 Le Mans, France

^2^ LUNAM Universités, CNRS, Université du Maine, IMMM UMR-CNRS 6283,

Av. O. Messiaen, 72085 Le Mans, France

^3^ LSPM, UPR-CNRS 3407, Université Paris Nord, Villetaneuse, France

*Corresponding authors: [vitali.goussev@univ-lemans.fr](mailto:vitali.goussev@univ-lemans.fr), [zerr@univ-paris13.fr](mailto:zerr@univ-paris13.fr)

**Supplementary Note 1**

**On the duration of the photo-generated acoustic pulse and ultimate in-depth spatial resolution of TDBS**

In depth-profiling of the spatially inhomogeneous media the in-depth spatial resolution of TDBS is known to be limited either by the spatial length of the coherent acoustic pulse or by restrictions introduced by specific signal processing techniques^1,2^. In our experiments the coherent picosecond acoustic pulse is predominantly generated by the thermo-elastic mechanism in the iron foil, because of the negligible energy transport from iron into ice caused by weak thermal diffusivity of ice in comparison with that of iron^3^. As the duration of the picosecond blue pump laser pulse (1.9 ps at FWHM) exceeds the characteristic time of the longitudinal acoustic wave propagation across the region of the absorbed optical energy release in iron, the duration of the photo-generated acoustic pulse is controlled by the duration of the laser pulse^4^. Note that the depth of lattice heating in Fe does not importantly broadened relative to the depth of the blue light penetration, because of the relatively strong coupling of the electrons and the phonons in Fe. Even for the overestimated depth of the energy release zone, 15 nm at FWHM, the time of sound propagation across it with the velocity of $8.4\cdot10^{3}$ m/s Ref.^5^ is shorter than the duration of the laser pulse. So the length of the coherent acoustic pulse launched into the ice sample near its interface with the Fe foil is estimated to be of about 30 nm at pressures of the experiment. This provides the best possible in-depth spatial resolution unless the amplitude of the launched pulse is so strong that the nonlinear acoustic effects could lead to weak shock front formation. In the latter case, which is actually not realized in our experiments, the spatial resolution could be controlled by the width of the shock front, which could be shorter than the width of the initially launched acoustic pulse^6,7^. In our linear case the situation is, in a certain sense, opposite to the case of the nonlinear acoustics. Due to the preferential absorption and scattering of high frequency components, the initially launched coherent acoustic pulse is continuously broadening along its propagation path. However, the abrupt variations in the Brillouin oscillations amplitude and frequency observed experimentally by passing the ice/diamond interface (Figs. 1 (c) and 2 (a)) indicate that the acoustic pulse is shorter than the Brillouin period even when arriving at the end of the ice sample. Thus, the duration of the acoustic pulses does not influence the spatial resolution of the images obtained in the present Communication provided the moving time windows are equal to or larger than the Brillouin period.

**Supplementary Note 2**

**On the role of the induced optical anisotropy**

In optically anisotropic grains the light experiences double or triple refraction and the optical rays, characterized by different refractive indexes, propagate after refraction with different velocities, which in addition depend on the direction of rays propagation relative to crystallographic axes^8,9^. However the ices VII and X of our interest here are cubic and, thus, optically isotropic. Consequently, the variations of the refractive index *n* could be caused in our samples only by anisotropy induced by nonhydrostatic stress component, i.e., due to uniaxial loading^10^. Strong induced anisotropy could potentially cause splitting of the Brillouin frequency peak in TDBS experiments conducted with linearly polarized light^11^. The splitting effect in polarized probe light is replaced by broadening and shift of the Brillouin frequency line in our experiments conducted with circular polarized light. Note that even under uniaxial loading cubic crystals in general become biaxial^8^ and exhibit triple refraction. An order-of-magnitude estimates demonstrate that induced optical anisotropy is negligible in our experiments. For example, in the experiments we consider as conducted at 84 GPa, this pressure was measured in the centre of the DAC where the data were accumulated, while at the edge of the ice sample at the distance *R=D*/2=45 *μm* from the centre the measured pressure was by $\Delta P=$4 GPa lower. This suggests presence of a uniaxial stress component parallel to the load axis of the DAC which value *t* can be estimated from $t\approx H(\Delta P/R)$, e.g. Ref.^12^ where the thickness *H* of the sample is 13.5 *μm*. In the considered case we obtained $t\approx$ 1 GPa. The characteristic value of the photo-elastic (acousto-optic) parameter $\partial n/\partial P$can be estimated from the experimental curve $n=n(P)$in Fig. 3 of Ref. 25 by $\partial n/\partial P\approx10^{-3}$ 1/GPa at 84 GPa. Thus, an order-of-magnitude estimate for the possible difference in the refractive indexes of different optical rays in nonhydrostatically compressed H_2_O ice is $\Delta n\propto(\partial n/\partial P)t\approx10^{-3}$. For the refractive index of $n\approx1.8$, estimated from the data in Ref.^13^ at *P*=84 GPa for the probe wavelength of 808 nm, this results in $\Delta n/n\propto5\cdot10^{-4}$, indicating that the variations of the refraction index in equation (1) is largely negligible. However, in view of the complexity of the phenomena of stress-induced anisotropy and of the lack of data on the photoelastic tensor of H_2_O ice VII and X, we conducted additional test experiments with variable linear polarisation of the probe beam. In Fig. 5 (a) we present three dependences on time of the Brillouin frequency, which are extracted from TDBS signals detected at 50 GPa in the same position of the sample but using instead of circularly polarized probe light linearly polarized light of three different polarizations rotated in steps of 60 degrees. The resolution is determined by a 133 ps FWHM duration of the Hanning temporal window chosen for the Fourier transform. The results in Fig. 5 (a) indicate that in our experimental system there are polarization-dependent effects. However, there could be different physical phenomena contributing to the variation of the Brillouin frequency with the polarization of probe light in Fig. 5 (a). The experimentally observed shifts in Brillouin frequency could be attributed to the stress-induced optical anisotropy of cubic ice only under the conditions that only the polarization of the probe light incident on the surface of the ice is changing, when we rotate its polarization outside the DAC. Only in this case the experiments provide direct access to the refractive indexes of the different probe rays in cubic ice with stress-induced anisotropy, because the only variable parameter influencing the detected Brillouin frequency will be the distribution/repartition of probe light among different rays interacting with the acoustic pulse. However, in general, this ideal condition is difficult to achieve because, firstly, the position of light beam incident on the diamond, and consequently on the ice, could be slightly shifted when optical elements are used to rotate its polarization and, secondly, the position of the probe, the shape of the light focus and the polarization of the probe on the ice surface all could be influenced by polarization-dependent optical phenomena in diamond, caused by its natural optical anisotropy and important induced optical anisotropy due to strongly nonuniform stress generated in the uniaxially loaded anvils^14-16^. For example, the shift of the probe focus and modification of its shape on the surface of the ice could cause the shift of the detected Brillouin frequency even in case of negligible induced optical anisotropy of cubic ice if the photo-generated acoustic pulse propagates simultaneously across just two or more crystallites with different sound velocities along acoustic pulse propagation direction. In this case rotation of probe light polarization incident on the DAC could cause the redistribution of the probe light incident on the different parts of the acoustic beam cross section and to possible modification of the Brillouin frequency, because different cross sections of the acoustic beam are propagating with different velocities. It is worth mentioning, that stresses in the diamond anvils cause optical anisotropy of the anvils even close to their symmetry axis. They were reported to result, for example, in distortion/transformation of the initially linearly polarised laser light into elliptical^17^. Although currently we are not able to identify precisely the physical reasons of the polarization-dependent effect reported in Fig. 5 (a), we believe this experimental test clearly indicates the weakness of the effect. Relative changes of Brillouin frequency in Fig. 5 (a) do not exceed 2 %. Thus, variations of the refractive index in equation (1) cannot be responsible for the 20% variations in the Brillouin frequency revealed in Figs. 2 and 4 and could be neglected when using equation (1) for the estimates of the acoustic velocity.

**Supplementary Note 3**

**On the role of the acoustic rays refraction**

The following considerations indicate that the dependence of the BS frequency on $\cos\theta$ can be neglected in equation (1) in comparison with the dependence on the sound velocity. When the acoustic wave is incident obliquely from an elastically isotropic medium (1) on its interface with an elastically isotropic medium (2) the direction of the transmitted wave shifts from the direction of the incident wave by a certain angle $\theta'$. If we denote by $\upsilon_{1,2}$ the sound velocities in the first and the second medium, respectively, then in the case $\upsilon_{1}>\upsilon_{2}$ the maximum refraction takes place at grazing incidence (), while in the case $\upsilon_{1}<\upsilon_{2}$ it takes place at critical angle of incidence $\theta_{cr}=\arcsin(\upsilon_{1}/\upsilon_{2})$ () . It is worth noting that refraction angle relative to the initial propagation direction, accumulated through successive incidence at different angles on multiples interfaces, can’t exceed the maximum refraction angle of single scattering. The maximum refraction angle obviously diminishes with diminishing difference between the acoustic velocities in the contacting materials. In the limiting case of small difference of sound velocities, denoting $\upsilon_{1}=(\upsilon_{1}+\upsilon_{2})/2+(\upsilon_{1}-\upsilon_{2})/2\equiv\left\langle\upsilon\right\rangle+\Delta\upsilon$ and $\upsilon_{2}=\left\langle\upsilon\right\rangle-\Delta\upsilon$, with $\left|\Delta\upsilon\right|/\left\langle\upsilon\right\rangle<<1$, we estimate . This clear asymptotic result indicates that in polycrystalline samples with a weak elastic anisotropy of grains, i.e., with $\left|\Delta\upsilon\right|/\left\langle\upsilon\right\rangle<<1$, the contribution to relative variations of the Brillouin frequency $\Delta f_{B}/\left\langle f_{B}\right\rangle$ in Eq. (1), which is coming from the directional variations in the sound velocity and is proportional to $\Delta\upsilon/\left\langle\upsilon\right\rangle$, dominates over the contribution due to possible refraction of sound, which is proportional to . In these estimates, for the values of $\upsilon_{1,2}$ we should use the values of the maximum and the minimum values of the direction-dependent sound velocity in the microcrystals. From the experimental data on the elastic moduli of H_2_O ice at 40 GPa presented in Ref.^18^ we estimate that the maximum longitudinal velocity (in [111] direction) is about 20% higher than the minimum longitudinal velocity (in [100] direction). This provides $\left|\Delta\upsilon\right|/\left\langle\upsilon\right\rangle\leq0.1$ and indicates that the contribution of variations in $\theta$ to the variations in the Brillouin frequency in equation (1) is at the level below 1%. Although in accordance with Ref.^18^ the anisotropy increases with increasing pressure, we believe that even in our experiments, which are conducted at higher pressures, the variations in the Brillouin frequency from grain to grain are largely dominated by possible changes in the sound propagation velocity and not in the sound propagation direction. In fact in our experimental results in Fig. 3, revealing large-scale texturing both at 57 GPa and 84 GPa, the maximum changes of the Brillouin frequency relative to the average level are about 10% ($\left|\Delta f_{B}\right|/\left\langle f_{B}\right\rangle\leq0.1$) and then $\Delta(\cos\theta)/\left\langle\cos\theta\right\rangle\leq0.01$. Even when superposing the short-scale texturing revealed in Fig. 4 (a) and (b) with the large scale texturing in Fig. 3 (b) the maximum changes of the Brillouin frequency relative to the average level in Fig. 4 (c) are about 20% ($\left|\Delta f_{B}\right|/\left\langle f_{B}\right\rangle\leq0.2$, $\Delta(\cos\theta)/\left\langle\cos\theta\right\rangle\leq0.04$). Consequently, in accordance with above presented estimates, the dependence on $\cos\theta$ in Eq. (1) is largely dominated by the dependence on the acoustic velocity.

**Supplementary Note 4**

**On the amplitude of the TDBS signal**

The following considerations indicate the dominant role of the photo-elastic and elastic anisotropy of cubic ice in the experimentally observed variations of the TDBS signal amplitude by propagation through the sample. In principle, the non-monotonous changes in the amplitude of the oscillations revealed in TDBS (Fig. 1 (c)) could be caused by beatings/interference between two or more oscillations at different frequencies. Simultaneous detection of several frequencies by TDBS would be expected if, for example, the elastically isotropic opto-acoustic generator, Fe-foil in our case, launches in elastically anisotropic H_2_O ice not only the quasi-longitudinal acoustic pulse, which currently dominates in all our signals, but also one or two quasi-traverse acoustic pulses, propagating at velocities importantly slower than the quasi-longitudinal one and resulting in TDBS signals at very different frequencies. Generation of quasi-shear acoustic pulses by mode conversion of the longitudinal acoustic pulses at the interface between isotropic light absorbing photo-acoustic generator and anisotropic transparent elastic media was earlier reported^19^, and beatings in the TDBS signals, caused by the interference of the Brillouin oscillations induced by quasi-longitudinal and quasi-shear waves, were observed^19-23^. Our experiments currently indicate that either the mode conversion of the longitudinal pulse into quasi-shear pulses at the Fe/ice interface and the mode conversion of quasi-longitudinal pulse into quasi-shear pulses at the interfaces between differently oriented crystallites are inefficient or the photo-elastic detection of quasi-shear waves is inefficient, or both. Several frequencies can be potentially found in TDBS signals in optically anisotropic media because of splitting of the probe laser beam in such media into two or three independent beams, which are characterized by different values of the refractive indexes^11^. However, cubic H_2_O ice is optically isotropic and our additional testing measurements have demonstrated that optical anisotropy, induced by possible deviation of mechanical loading of crystallites in DAC from the hydrostatic conditions, is negligibly small (see Fig. 5). It is also worth mentioning here that the period of possible beatings in TDBS signal amplitude, when two oscillations at different frequencies, *f*_1_ and *f*_2_, are contributing to it, is equal to 1/|*f*_1_*-f*_2_|. Thus, even if we estimate the difference between frequencies corresponding to ordinary and extraordinary light, |*f*_e_-*f*_o_|, by double frequency shift detected in Fig.5, i.e., about 2 GHz, the expected beating period will be 500 ps, i.e., much longer than the time interval where birefringence is observed in this Figure. This is an additional argument in favor of the conclusion that the observed non-monotonous variations of TDBS signal amplitude are not caused by optical anisotropy.

Finally, the detection of several frequencies could be expected in polycrystalline samples if the photo-generated acoustic pulse and the probe light propagate simultaneously across several differently oriented crystallites. This could be possible not only if the diameters of the acoustic and probe laser beams are importantly larger than the characteristic dimension of the grains in lateral direction, i.e., in the plane parallel to the surface of the photo-generator, but also in the case when they are narrower than the individual grains but propagate along an interface of two grains, which is inclined relative to sound propagation direction, or they propagate along the interface separating larger groups of similarly oriented crystallites. In our experiments reported here the diameters of the acoustic and the probe beams are exceeding the estimated dimensions of the individual crystallites, so the first and the third of the mentioned situations seem to be more relevant. However, in both these cases the situations when the cross section areas of the acoustic pulse in two differently oriented domains of ice are close in magnitude, leading to two oscillations of comparable amplitudes and to possibility of important beatings, are rear. In fact, in the experimental data presented in Fig. 3 the signal processing has not revealed two frequency peaks of comparable amplitudes along the complete paths of the photo-generated acoustic pulses.

The above discussion leads to the conclusion that non-monotonous variations of the TDBS signal amplitude in time are not manifestation of beatings/interference among different frequency components of the signal. They are rather the second, in addition to Brillouin frequency variation, direct manifestation of disorientation of grains or group of grains in our polycrystalline H_2_O sample.

**Supplementary References**

1. Lomonosov, A. M. et al. Noncontact subsurface investigations of mechanical and optical properties of nanoporous low-*k* material thin film. *ACS Nano* **6**, 1410 (2012).

2. Mechri, C. et al. Depth-profiling of elastic inhomogeneities in transparent nanoporous low-k materials by picosecond ultrasonic interferometry. *Appl. Phys. Lett.* **95**, 091907 (2009).

3. Nikitin, S. M. et al. Directivity patterns and pulse profiles of ultrasound emitted by laser action on interface between transparent and opaque solids: Analytical theory. *J. Appl. Phys.* **115**, 044902 (2014).

4. Gusev, V. & Karabutov, A. *Laser Optoacoustics* (AIP, New York, 1993).

5. Fiquet, G., Badro, J., Guyot, F., Requardt, H. & Krisch, M. Sound velocities in iron to 110 gigapascales. *Science* **291**, 468 (2001).

6. Gusev, V. Detection of nonlinear picosecond acoustic pulses by time-resolved Brillouin scattering. *J. Appl. Phys.* **116**, 064907 (2014).

7. Klieber, C., Gusev, V. E., Pezeril, T. & Nelson, K. A. Nonlinear acoustics at GHz frequencies in a viscoelastic fragile glass former. *http://arxiv.org/abs/1403.3222* (2014).

8. Born, M. & Wolf, E. *Principles of Optics* (Pergamon Press, London, 1970).

9. Ramachandran, G. N. & Ramaseshan, S. in *Encyclopedia of Physics* (ed. Flugge , S.) 1-217 (Springer, Berlin, 1961).

10. Singh, A. K. The lattice strains in a specimen (cubic system) compressed nonhydrostically in an opposed anvil device. *J. Appl. Phys.* **73**, 4278 (1993).

11. Lejman, M. et al. Giant ultrafast photo-induced shear strain in ferroelectric BiFeO_3_. *Nat. Commun.* **5**, 4301 (2014).

12. Eremets, M. *High pressure experimental methods* (Oxford University Press, Oxford, 1996).

13. Zha, C.-S., Hemley, R. J., Gramsch, S. A., Mao, H.-K. & Bassett, W. A. Optical study of H_2_O ice to 120 GPa: dielectric function, molecular polarizability, and equation od state. *J. Chem. Phys.* **126**, 074506 (2007).

14. Bruno, M. S. & Dunn, K. J. Stress analysis of a beveled diamond anvil. *Rev. Sci. Instrum.* **55**, 940-943 (1984).

15. Hanfland, M. & Syassen, K. A Raman study of diamond anvils under stress. *J. Appl. Phys.* **57**, 2752-2756 (1985).

16. Merkel, S., Hemley, R. J., Mao, H.-K. & Teter, D. M. in *Science and Technology of High Pressure, Proceedings of the AIRAPT -17* (eds. Manghnani, M. H., Nellis, W. J. & Nicol, M. F.) 68-73 (University Press, Hyderabad, 2000).

17. Ninet, S., Datchi, F., Saitta, A. M., Lazzeri, M. & Canny, B. Raman spectrum of ammonia IV. *Phys. Rev. B* **74**, 104101 (2006).

18. Zha, C.-S., Mao, H.-K., Hemley, R. J. & Duffy, T. S. Recent progress in high-pressure Brillouin scattering: olivine and ice. *Rev. High Pressure Sci. Technol.* **7**, 739 (1998).

19. Bienville, T. & Perrin, B. in *Proceedings of the World Congress on Ultrasonics 2003,* 813-816 (Paris, 2003).

20. Matsuda, O., Wright, O., Hurley, D., Gusev, V. & Shimizu, K. Coherent shear phonon generation and detection with ultrashort optical pulses. *Phys. Rev. Lett.* **94**, 095501 (2004).

21. Matsuda, O., Wright, O. B., Hurley, D. H., Gusev, V. E. & Shimizu, K. Coherent shear phonon generation and detection with picosecond laser acoustics. *Phys. Rev. B* **77**, 224110 (2008).

22. Ruello, P. et al. Photoexcitation of gigahertz longitudinal and shear acoustic waves in BiFeO_3_ multiferroic single crystal. *Appl. Phys. Lett.* **100**, 212906 (2012).

23. Wen, Y.-C., Ko, T.-S., Lu, T.-C., Kuo, H.-C. & Chyi, J.-I. S., C.-K. Photogeneration of coherent shear phonons in oriented wurtzite semiconductors by piezoelectric coupling. *Phys. Rev. B* **80**, 195201 (2009).
